# The prevalence of human papillomavirus in pediatric tonsils: a systematic review of the literature

**DOI:** 10.1186/s40463-018-0255-1

**Published:** 2018-01-30

**Authors:** Monika Wojtera, Josee Paradis, Murad Husein, Anthony C. Nichols, John W. Barrett, Marina I. Salvadori, Julie E. Strychowsky

**Affiliations:** 1 Schulich School of Medicine and Dentistry, Western University, 1151 Richmond St, London, N6A 5C1 ON Canada; 2 Department of Otolaryngology-Head and Neck Surgery, Victoria Hospital B3-400, 800 Commissioners Rd E, London, N6A 5W9 ON Canada; 3 Department of Paediatrics, Children’s Hospital at London Health Sciences Centre, 800 Commissioners Rd E, London, ON N6A 5W9 Canada

**Keywords:** Pediatric, Human papillomavirus, HPV, Tonsils

## Abstract

**Background:**

HPV-related head and neck cancer rates have been increasing in recent years, with the tonsils being the most commonly affected site. However, the current rate of HPV infection in the pediatric population remains poorly defined. The objective of this study was to systematically review and evaluate the prevalence and distribution of HPV in the tonsils of pediatric patients undergoing routine tonsillectomy.

**Methods and Results:**

The literature was searched using PubMed, EMBASE, Scopus, CINAHL, Cochrane Library, and ProQuest Dissertations & Theses Global databases (inception to December 2017) by two independent review authors. Inclusion criteria included articles which evaluated the prevalence of HPV in a pediatric cohort without known warts or recurrent respiratory papillomatosis, those which used tonsil biopsy specimens for analysis, and those with six or more subjects and clear outcomes reported. Eleven studies met the inclusion criteria. Using the Oxford Clinical Evidence-based Medicine (OCEBM) guidelines, two reviewers appraised the level of evidence of each study, extracted data, and resolved discrepancies by consensus. The systematic review identified 11 articles (*n* = 2520). Seven studies detected HPV in the subject population, with prevalence values ranging from 0 to 21%. The level of evidence for all included studies was OCEBM Level 3.

**Conclusions:**

HPV may be present in pediatric tonsillectomy specimens; however, the largest included study demonstrated a prevalence of 0%. Future testing should be performed using methods with high sensitivities and specificities, such as reverse transcript real-time PCR or digital droplet PCR.

## Background

Human papillomavirus (HPV) is a DNA virus capable of infecting skin or stratified epithelial cells. There are over 100 different types of HPV, of which at least 13 are cancer-causing types deemed ‘high-risk’; the most common of these being HPV 16 and 18. The remaining ‘low-risk’ types cause non-cancerous lesions and diseases, with HPV 6 and 11 infections occurring most frequently. While sexual transmission is the primary method of HPV infection, vertical transmission, auto-inoculation, and other forms of horizontal transmission have also been considered.

While most HPV infections remain subclinical and are eventually eliminated by the immune system, some cases may persist [1]. In children, this persistence commonly presents as recurrent respiratory papillomatosis (RRP), associated with HPV 6 or 11, or anogenital warts, associated with multiple HPV subtypes. In adults, presentations range from benign skin and anogenital warts due to low-risk subtypes to more serious cervical, anal, and head and neck cancers secondary to high-risk subtypes. The vast majority HPV-related head and neck cancer arises in the oropharynx, with the tonsils being the most commonly affected site [2–4]. Despite the decreasing frequency of tobacco- and alcohol-related oropharyngeal squamous cell cancer (OPSCC), the prevalence of HPV-positive cases has been progressively increasing and is now over 70%, due to rising rates of oral infection with HPV [5–9]. Of note, HPV 16 accounts for the majority of cases (> 90%) [10]. Since 6.9% of the adult population has been found to have detectable HPV at any given point in time [5], it is important to delineate both when tonsillar HPV infection is first acquired and how long it remains latent by evaluating its prevalence in the pediatric population. However, the rate of HPV infection in the tonsillar tissue of the pediatric population remains poorly defined. In this manuscript, we endeavor to carry out a systematic review of the prevalence of HPV in pediatric patients undergoing routine tonsillectomy.

## Methods

This review was conducted in accordance with the Preferred Reporting Items for Systematic Reviews and Meta-Analyses (PRISMA) 2009 guidelines [11].

### Literature search strategy

A comprehensive literature search was performed using PubMed, EMBASE, Scopus, CINAHL, Cochrane Library, and ProQuest Dissertations & Theses Global databases on December 10, 2017. Similar search strategies were applied to search each database. The electronic database search combined disease-specific terms (human papillomavirus, HPV) with anatomic-specific terms (tonsil, throat, pharynx, adenoid, palatine, tubal, lingual) and pediatric-specific terms (pediatric, paediatric, children, kid, youth, teen, preteen, minor, juvenile, virgin, pre-pubescent, prepubescent, boy, girl, toddler, baby, babies, infant, neonate, newborn). Initially, to ensure the consideration of all relevant published articles in the initial search, the search was not limited in terms of publication date, study design, or language of publication. Relevant articles and abstracts were selected and reviewed, and their reference lists were further searched for additional publications. Next, all studies with abstracts or full articles written in the English language were considered for inclusion.

### Study selection criteria

Articles were assessed for eligibility and included if they reported a prevalence of HPV in pediatric tonsils (age < 18 years old), regardless of study design or publication date. Articles were excluded if they did not report results separately for the pediatric population, if they analyzed specimens from anatomic sites other than the palatine tonsils, or if non-biopsy methods of tonsillar testing were used. Where studies analyzed tonsil specimens from adults as well as children, only the pediatric data was used in our analysis. If discerning between the two was not possible, the study was excluded.

To detect tonsillar HPV, studies that utilized tonsillectomy specimens as a sampling method were used. The detection of HPV using swabs of the oral mucosa, tonsillar mucosa, and nasopharynx is inconsistent with the results attained by biopsy, and therefore these sampling methods were excluded. It is theorized that HPV tends to localize to the epithelium of tonsillar crypts, which would account for the unreliability of tonsillar swabs as a method of detection [12]. In cases where oropharyngeal scrapings were used as opposed to tonsillar scraping or biopsy, that study or portion of the study was excluded [13].

Articles in a foreign language were included as long as an abstract was available in the English language and if the abstract described the method of specimen acquisition and reported the prevalence of HPV [13, 14].

### Data analysis

The methodological quality of identified studies was appraised using the Oxford Center for Evidence-Based Medicine (OCEBM) 2011 Levels of Evidence [15] and the Newcastle-Ottawa Scale modified for cross-sectional studies [16]. Relevant data was extracted from included studies following tables developed a priori by two independent review authors (M.W. and J.S.). Disagreement was resolved by consensus. Data collected included the country of study, number of patients, age range, mean age, reported prevalence of HPV, testing method, types of HPV tested, and types of HPV detected. Qualitative and quantitative synthesis of results was performed when applicable. A pooled proportion of the prevalence of HPV in pediatric tonsils was considered.

## Results

**Fig. 1:**
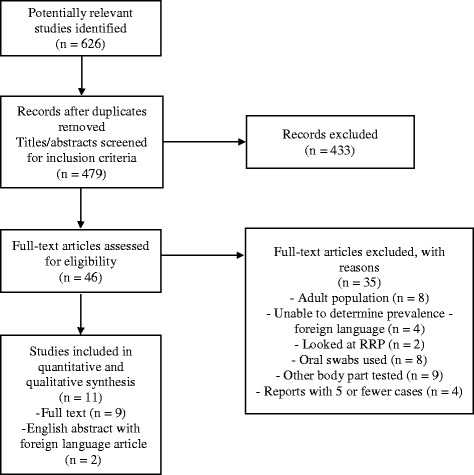
Flow diagram outlining search strategy

Six hundred twenty-six studies were identified during the initial literature search (Fig. 1). Four hundred seventy-nine studies remained for consideration after duplicate publications were removed. The titles and abstracts of these studies were screened for the inclusion and exclusion criteria, and 433 studies were eliminated because of excluded records. The remaining 46 full-text articles were reviewed in their entirety. Eleven studies with a total of 2520 patients met the inclusion criteria and were included in the analysis. Among these, nine were full-text articles in English while two were abstracts in English with the full-text articles in another language.

**Fig. 2:**
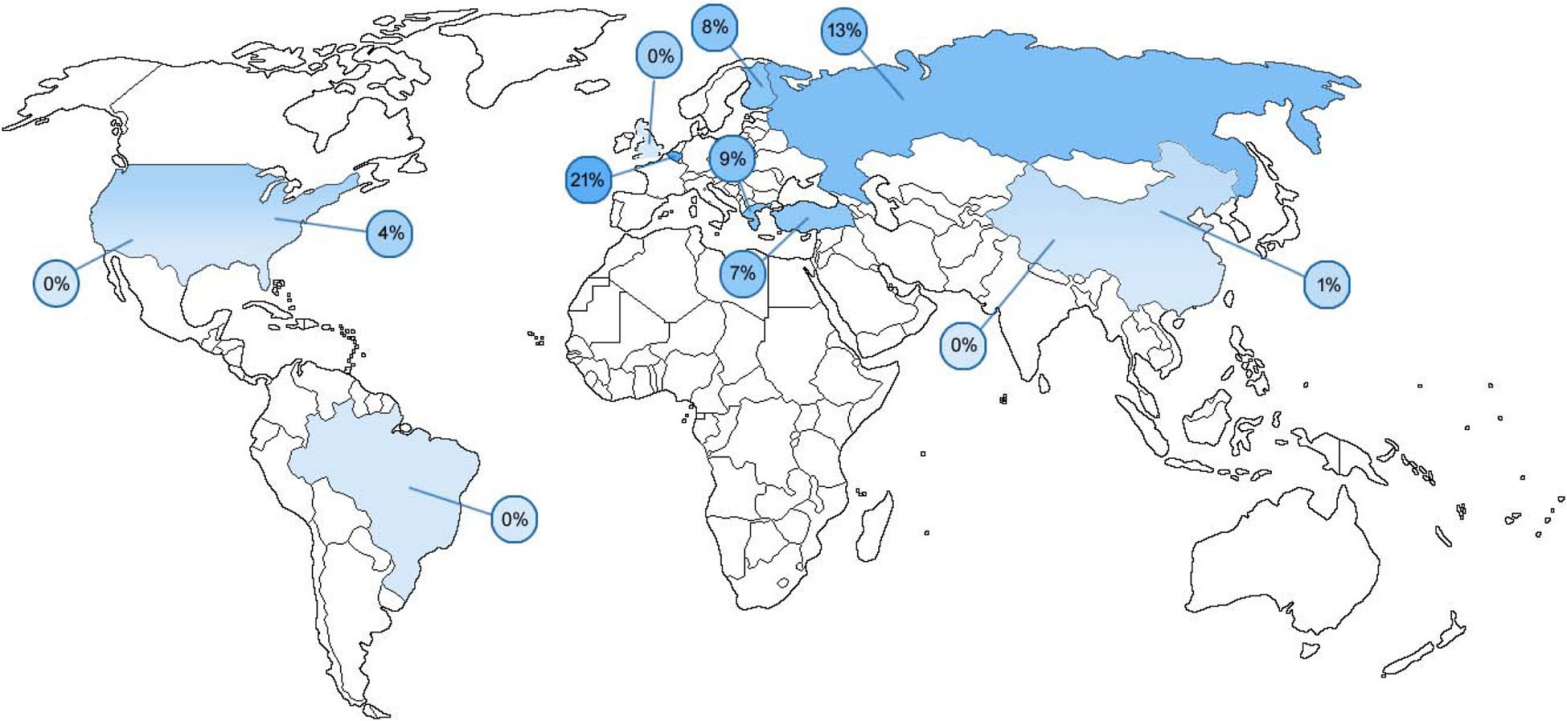
Geographical distribution of HPV infection in pediatric tonsils. A darker shade of blue represents a higher prevalence (≥5%), while a lighter tone represents a lower prevalence (< 5%). USA – 0%^17^, 4%^23^, United Kingdom – 0%^18^, China - 0%^19^, 1%^14^, Belgium – 21%^20^, Turkey – 7%^21^, Russia – 13%^13^, Greece – 9%^22^, Brazil – 0%^24^, Finland – 8%^25^

Study and patient characteristics are reported in Table 1. The number of patients enrolled in each study ranged from 8 to 1670. HPV was detected in seven studies, and the prevalence of HPV ranged from 0 to 21%. The geographic distribution is illustrated in Fig. 2. The HPV subtypes that were detected varied: subtype 6 (two studies) [14, 17], 11 (three studies) [14, 17, 18], 16 (four studies) [17, 19–21], and 31 (one study) [17]. There was variability in testing methods and HPV subtypes tested, with the majority of studies using conventional PCR with broad-spectrum probes (8/11 studies, 72%), with four studies following up this initial screen with type-specific probes. Three studies used real-time PCR (qPCR), and none used reverse-transcriptase qPCR (RT-qPCR). Reporting and subgroup analysis of the ages and genders of HPV-positive samples was considered, but was not possible as most included studies did not provide these details.

**Table 1:** Summary of included studies

First Study Author & Year of Publication^a^	Country	# of Patients	Age Range (Years) [mean]	Prevalence of HPV^a^	Testing Method	Types of HPV Tested	Types of HPV Found
Cockerill, CC (2016) [25] [full text] ^b^	USA	129	1–12 [NR]	0%	Biopsy + swabs + Roche Cobas Amplicor test qPCR & E6/E7 Gene-Probe Aptima HPV test	16, 18, 31, 33, 35, 39, 45, 51, 52, 56, 58, 59, 66, 68	None
Palmer, E (2014) [27] [full text]	United Kingdom	1670	0–18 [NR]	0%	Biopsy + GP5+/6+ primer PCR enzyme immunoassay + PCR E6 gene targeting for HPV16	20 types (unspecified), 14 high-risk and 6 low-risk	None
Xue, XC (2014) [26] [full text]	China	42	3–12 [6.8]	0%	Biopsy + MY09/11 primer qPCR	6, 11, 16, 18, 26, 31, 33–35, 39, 40, 42–45, 51–59, 61, 66–73, 81–84	None
Sun, YF (2012) [abstract] [article in Chinese] [14] [full text]	China	177	NR[NR]	1%	Biopsy + PCR (unspecified)	NR	6, 11
Duray, A (2011) [19] [full text]	Belgium	42	0–15 [NR]	21%	Biopsy + GP5+/GP6+ primer PCR + E6/E7 type-specific qPCR for multiple HPV subtypes	6, 11, 16, 18, 31, 33, 35, 39, 45, 51, 52, 53, 56, 58, 59, 66, 68	16, NR
Baloglu, H (2010) [17] [full text]	Turkey	165	5–21 [11.9]	7%	Biopsy + MY/GP PCR + E6/E7 type-specific PCR	6, 11, 16, 18, 31, 33, 35, 39, 42, 43, 44, 45, 51, 52, 56, 58, 59, 66, 68	6, 11, 16, 31
Soldatskiĭ, IuA (2009) [abstract] [article in Russian] [13]	Russia	8	2–14 [6.8]	13%	PCR	6, 11, 16, 18, 31, 33	NR
Mammas, IN (2006) [20] [full text]	Greece	64	2–14 [7.1]	9%	Biopsy + GP5+/6+ primer PCR + type-specific PCR for multiple HPV subtypes	11, 16, 18, 33	16, NR
Sisk, J (2006) [18] [full text]	USA	50	3–12 [NR]	4%	Biopsy + MY09/11 primer PCR	NR	11
Ribeiro, KM (2006) [37] [full text]	Brazil	100	2–13 [NR]	0%	Biopsy + MY09/11 primer PCR	NR	None
Chen, R (2005) [21] [full text]	Finland	73	1–16 [8.3]	8%	Biopsy + GP5+/6+ & MY09/11 primer PCR + type-specific testing	NR	16

*HPV* human papillomavirus, *NR* not reported in the study or for the tonsillectomy portion

^a^ Articles published in English unless otherwise specified

^b^ This study only assessed high-risk HPV subtypes. This was also the only study to report that some patients had been vaccinated against HPV; 6 (4.7%) of patients had received the vaccine

The OCEBM level of evidence for all 11 included studies was Level 3. Quality assessment according to the Newcastle-Ottawa Scale modified for cross-sectional studies is reported in Table 2.

**Table 2:** Quality assessment using a version of the Newcastle-Ottawa modified for cross-sectional studies

Author (Year)	Selection (Maximum 4 Stars)	Comparability (Maximum 2 Stars)	Exposure (Maximum 3 Stars)	Total (Maximum 9 Stars)
Cockerill, CC (2016) [25]	^**^	^*^	^*^	^****^
Palmer, E (2014) [27]	^***^	^*^	^**^	^******^
Xue, X-C (2014) [26]	^**^	^*^	^**^	^*****^
Sun, YF (2012) [abstract] [article in Chinese] [14]	^**^	^**^	^*^	^*****^
Duray, A (2011) [19]	^**^	^*^	^*^	^****^
Baloglu, H (2010) [17]	^**^	^*^	^*^	^****^
Soldatskiĭ, IuA (2009) [abstract] [article in Russian] [13]		^*^	^*^	^**^
Mammas, IN (2006) [20]	^**^	^**^	^**^	^******^
Sisk, J (2006)[18]	^**^	^**^	^**^	^******^
Ribeiro, KM (2006)[37]	^**^	^*^	^*^	^****^
Chen, R (2005)[21]	^*^	^*^	^*^	^***^

Although a pooled statistical analysis was considered for the collected data, the variability in sample size, geographic distribution, HPV testing methods, and types of HPV tested would limit generalizability of possible pooled analyses. Due to this heterogeneity, a pooled analysis of the data was not performed.

## Discussion

To our knowledge, this is the first comprehensive analysis of the prevalence of HPV in pediatric tonsillar tissue. Based on this systematic review of the literature, the prevalence ranges from 0 to 21%.

### Study limitations

The wide variability in the prevalence of HPV between studies warrants discussion. While each of the studies used biopsy specimens for testing, the majority used conventional PCR and broad-spectrum GP5+/6+ or MY09/11 primers. Conventional PCR has been noted to exhibit poor specificity and consequently a high false positive rate when testing for HPV, which may explain some of the high prevalence findings reported [22]. qPCR is more accurate, with an improved ability to discriminate between positive and negative samples based the cycle threshold [23]. However, the gold standard is RT-qPCR, which amplifies signals based on the presence of viral transcripts and demonstrates the expression of early viral proteins, as the significance of the presence of HPV DNA in the absence of viral expression is unclear [23]. A newer technique is digital droplet PCR (ddPCR), which may prove to be a more sensitive technique for HPV viral load quantification [24]. The unreliability of conventional PCR and lack of controls in some studies may explain the differences observed. Only three studies [20, 25, 26] used qPCR for analysis, and none employed RT-qPCR or ddPCR.

It is also of value to note that although the overall prevalence of HPV ranged from 0 to 21%, the largest study [27], encompassing 1670 patients, was unable to detect the virus. In total, four studies found a 0% prevalence of HPV, comprising 1941 of this review’s 2520 patients. Studies with positive results were all completed on a smaller scale; the largest patient sample from a study with a positive result consisted of 177 patients, with a prevalence of only 1% [14], suggesting potential sampling or selection bias. It is also important to consider that in all cases, tonsillar hypertrophy and chronic tonsillitis were the indications for tonsillectomy. Although these conditions are not known to be related to HPV infection, it is possible that they may be confounders.

### Geographical considerations

**Fig. 3:**
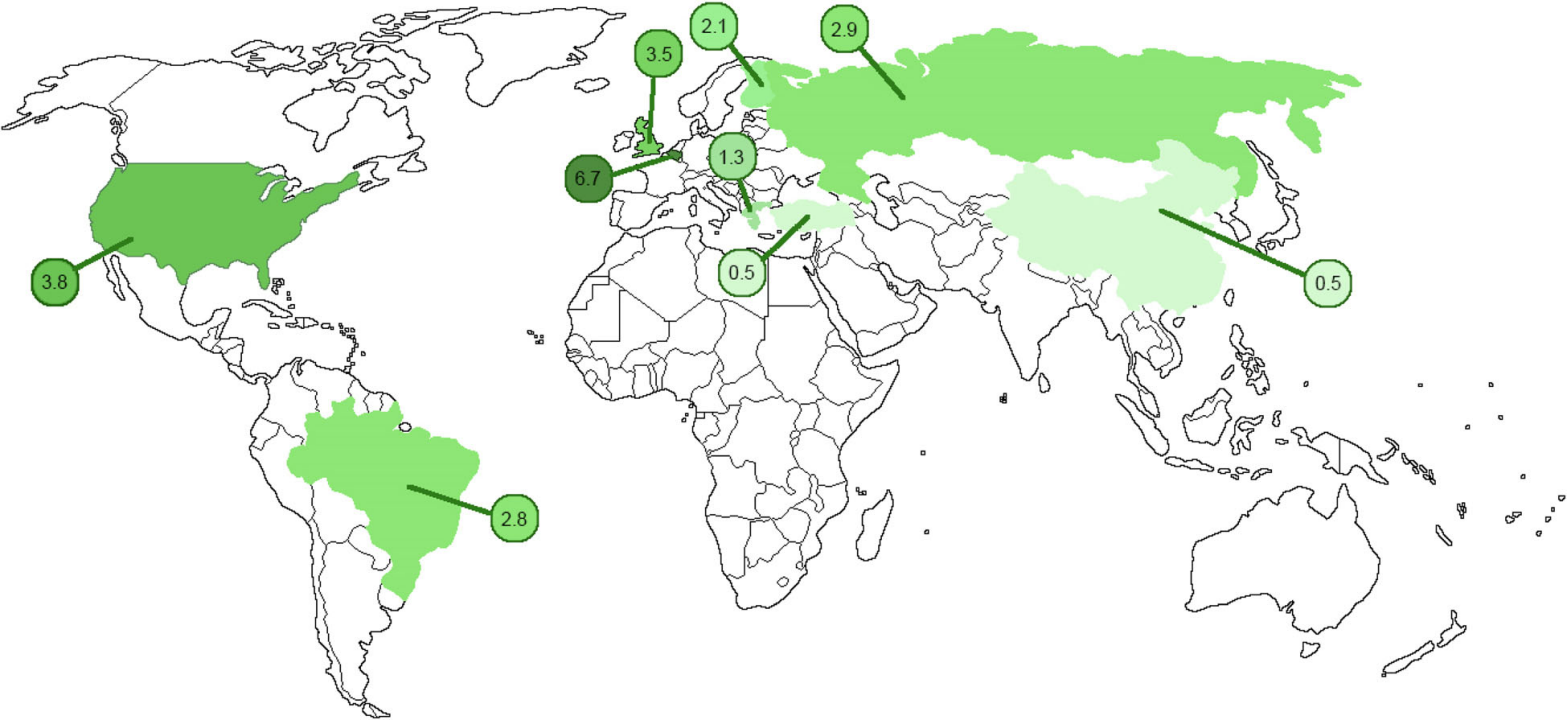
Geographical distribution of oropharyngeal cancer incidence rates per 100,000 worldwide. A darker shade of green represents rate higher than the worldwide average (≥2.0),^31^ while a lighter tone represents a lower rate (< 2.0%). Source: ^31^Ferlay J, Soerjomataram I, Ervik M, et al. Cancer incidence and mortality worldwide: IARC CancerBase No. 11. *GLOBOCAN*. 2012;1.0. Available from: http://globocan.iarc.fr, accessed on 22/06/2017. USA – 3.8^31^, United Kingdom – 3.5^31^, China – 0.5^31^, Belgium – 6.7^31^, Turkey – 0.5^31^, Russia – 2.9^31^, Greece – 1.3^31^, Brazil – 2.8^31^, Finland – 2.1^31^

An alternate possibility for the variation in prevalence of pediatric tonsillar HPV infection is that this infection has a geographical distribution, as illustrated in Fig. 2, with the highest prevalence in Belgium. HPV-related cervical lesions have been found to have a geographical distribution [28, 29], supporting the notion of such a pattern in tonsillar HPV. However, it does not appear as though worldwide oropharyngeal cancer incidence rates have a parallel distribution (Fig. 3) [30]. Country-specific prevalence rates could not be determined based on currently-available data. Of note, the highest rates for both pediatric HPV prevalence and oropharyngeal cancer incidence among the countries identified was Belgium; however, there is no obvious pattern beyond this. This lack of correlation could be simply because there is no relationship between pediatric tonsillar HPV infection and later adult oropharyngeal cancer; perhaps pediatric patients are more easily able to eliminate the infection, or prior HPV infection protects against future infection. Alternately, a correlation between childhood infection and adult cancer may exist, but be difficult to discern at this time due to the currently limited literature.

### Subtypes of Tonsillar HPV

Of the HPV subtypes identified among the patients included in this study (*n* = 28), HPV 6, 11, 16, and 31 were observed, with HPV 16 being the most prevalent (78.6%). High-risk HPV 16 is the subtype most frequently associated with OPSCC, with an average age of diagnosis of 61 years old [7]. This suggests that the subclinical HPV infection detected in childhood may be a pre-malignant lesion with a long-term course, representing a risk factor for the development of tonsillar cancer in adulthood. No studies reported long-term follow-up to determine the incidence of possible malignant transformation.

Whether this pediatric infection eventually progresses to symptomatic disease or cancer, remains dormant for the lifespan of the patient, or is eventually eliminated by the host’s immune system, is currently unknown. An interesting parallel is the pathogenesis of HPV infection in the cervix, wherein persistent and often asymptomatic HPV infection can lead to intraepithelial neoplasia and the eventual accumulation of mutations, resulting in cancerous invasion and metastasis [31]. A similar cycle may occur with tonsillar HPV infection, emphasizing the importance of early detection of HPV.

### HPV transmission and implications

The presence of HPV in the tonsils of pediatric patients suggests that consideration must be given for a mode of transmission other than sexual transmission. While sexual transmission through early sexual activity or sexual abuse remains a possibility [32–34], the detection of HPV in infants and in high rates among youth in some studies suggests that sexual exposure is unlikely to account for all cases. As such, vertical and horizontal transmission must also be considered.

### HPV vaccination

Both bivalent and quadrivalent vaccines against HPV are currently available on the world market, and a 9-valent vaccine has also been introduced in some areas. The bivalent vaccine is effective at preventing the high-risk HPV 16 and 18 subtypes, and the quadrivalent vaccine adds protection against HPV 6 and 11. As of 2014, 58 countries had introduced one or both versions of the HPV vaccine into their national immunization programs for girls, and countries such as Canada, the USA, Australia, and Austria also currently advocate its use in boys [22, 35].

Despite the availability of a publicly-funded vaccine against HPV infection in Canada, immunization rates for HPV remain far below those of other vaccine-preventable diseases [36]. This study calls into questions the preconception that HPV is solely a sexually-transmitted disease, and may be used to further vaccination campaigns in locations where stigma against vaccination for a sexually transmitted infection is still a barrier for larger-scale immunization campaigns.

## Conclusion

HPV 6, 11, 16, and 31 may be present in some pediatric tonsils, and their prevalence ranges from 0 to 21%. The largest study demonstrated a prevalence of 0%. A geographical distribution could account for these discrepancies, but does not appear to correlate with adult oropharyngeal cancer incidence rates. Whether these infections are a pre-malignant state, a transient infection, or a latent infection that will ultimately lead to malignant disease is yet to be determined. Further adequately powered studies utilizing qPCR, the gold-standard RT-qPCR, or the newer and more sensitive ddPCR for detection of HPV are warranted to determine the true prevalence of HPV in the pediatric population.

## Abbreviations

ddPCR: Digital droplet PCR
HPV: Human papillomavirus
OCEBM: Oxford clinical evidence-based medicine
OPSCC: Oropharyngeal squamous cell cancer
PRISMA: Preferred reporting items for systematic reviews and meta-analyses
qPCR: Real-time PCR
RRP: Recurrent respiratory papillomatosis
RT-qPCR: Reverse-transcriptase qPCR
